# Neurobehaviour between birth and 40 weeks’ gestation in infants born <30 weeks’ gestation and parental psychological wellbeing: predictors of brain development and child outcomes

**DOI:** 10.1186/1471-2431-14-111

**Published:** 2014-04-24

**Authors:** Alicia J Spittle, Deanne K Thompson, Nisha C Brown, Karli Treyvaud, Jeanie LY Cheong, Katherine J Lee, Carmen C Pace, Joy Olsen, Leesa G Allinson, Angela T Morgan, Marc Seal, Abbey Eeles, Fiona Judd, Lex W Doyle, Peter J Anderson

**Affiliations:** 1 Victorian Infant Brain Studies, Murdoch Childrens Research Institute, 4th Floor, Flemington Road, Parkville, Victoria 3052, Australia; 2 Department of Physiotherapy, School of Health Sciences, University of Melbourne, Level 7, Building 104, 161 Barry Street, Carlton, Victoria 3053, Australia; 3 Neonatal Services, Royal Women's Hospital, 7th Floor, Cnr Grattan Street and Flemington Road, Parkville, Victoria 3052, Australia; 4 Department of Paediatrics, University of Melbourne, Flemington Road, Parkville, Victoria 3052, Australia; 5 Department of Obstetrics and Gynaecology, 7th Floor, Cnr Grattan Street and Flemington Road, Parkville, Victoria 3052, Australia; 6 Melbourne School of Psychological Sciences, University of Melbourne, 12th Floor, Redmond Barry Building, Parkville, Victoria 3010, Australia; 7 Language and Literacy Group, Murdoch Childrens Research Institute, 5th Floor, Flemington Road, Parkville, Victoria 3052, Australia

**Keywords:** Preterm, Neurobehaviour, Magnetic resonance imaging, Neurodevelopment, Parent mental health, Parent–child interaction

## Abstract

**Background:**

Infants born <30 weeks’ gestation are at increased risk of long term neurodevelopmental problems compared with term born peers. The predictive value of neurobehavioural examinations at term equivalent age in very preterm infants has been reported for subsequent impairment. Yet there is little knowledge surrounding earlier neurobehavioural development in preterm infants prior to term equivalent age, and how it relates to perinatal factors, cerebral structure, and later developmental outcomes. In addition, maternal psychological wellbeing has been associated with child development. Given the high rate of psychological distress reported by parents of preterm children, it is vital we understand maternal and paternal wellbeing in the early weeks and months after preterm birth and how this influences the parent–child relationship and children’s outcomes. Therefore this study aims to examine how 1) early neurobehaviour and 2) parental mental health relate to developmental outcomes for infants born preterm compared with infants born at term.

**Methods/Design:**

This prospective cohort study will describe the neurobehaviour of 150 infants born at <30 weeks’ gestational age from birth to term equivalent age, and explore how early neurobehavioural deficits relate to brain growth or injury determined by magnetic resonance imaging, perinatal factors, parental mental health and later developmental outcomes measured using standardised assessment tools at term, one and two years’ corrected age. A control group of 150 healthy term-born infants will also be recruited for comparison of outcomes. To examine the effects of parental mental health on developmental outcomes, both parents of preterm and term-born infants will complete standardised questionnaires related to symptoms of anxiety, depression and post-traumatic stress at regular intervals from the first week of their child’s birth until their child’s second birthday. The parent–child relationship will be assessed at one and two years’ corrected age.

**Discussion:**

Detailing the trajectory of infant neurobehaviour and parental psychological distress following very preterm birth is important not only to identify infants most at risk, further understand the parental experience and highlight potential times for intervention for the infant and/or parent, but also to gain insight into the effect this has on parent–child interaction and child development.

## Background

Improving medical technologies are assisting younger and smaller preterm infants to survive. Very preterm infants (defined as born at <32 weeks’ gestation) are at risk of long-term neurodevelopmental problems including cognitive, motor and behavioural impairments [1]. The most immature infants are at the greatest risk for later neurodevelopmental deficits, yet it is these infants whose early neonatal neurobehavioural development we understand the least. In particular, there is a lack of knowledge surrounding early neurobehavioural trajectories between birth and term equivalent age. Nor do we currently understand the relationships that altered neurobehavioural trajectories may have with other important factors such as perinatal complications, cerebral structure, and later developmental outcomes [2].

Whilst many decades ago newborn infants were considered passive, research has shown that the brain, not just the spinal cord, is involved in the infant’s responses, and, more importantly, the infant’s brain is active from birth [3]. Neonatal neurobehavioural examinations have been developed that assess the integrity of an infant’s central nervous system [4] and can be used as a tool to identify infants at risk of developmental disabilities [2]. These examinations include traditional neurological assessment items such as reflexes, posture and tone along with behavioural assessment items such as the infant’s ability to regulate their own level of arousal and states, to habituate, to attend and orient [5]. Neurobehavioural development of preterm infants differs from that of term born infants at term equivalent age [6,7] and has been shown to be related to environmental and biological factors [8]. Importantly, these differences in neurobehavioural developmental at term in preterm children compared with term born children are associated with later motor, cognitive and behavioural difficulties [5,9] and contribute to neurodevelopmental impairments in school-age children born preterm. A recent review of neonatal neurobehavioural assessments for preterm infants assessed the validity of the available tools for assessing neurobehaviour from birth to term equivalent age and concluded that there are several tools that are predictive of development at age one and two years’ corrected age (CA) when used at term equivalent age and beyond [2,10,11]. However, there is a lack of evidence for the discriminative, evaluative and predictive validity of these tools when administered prior to term, limiting their use in clinical practice and research with preterm infants while in the Neonatal Intensive Care Unit (NICU). It is essential that health professionals can assess neurobehaviour from birth to (i) determine whether a very preterm infant is developing normally or abnormally, (ii) assess the effects of interventions and exposure to perinatal variables, and (iii) predict whether the infant may have long term developmental problems, so that appropriate interventions can be commenced immediately. Preliminary evidence suggests that neurobehaviour in the ex-utero environment matures rapidly during the neonatal period in association with cerebral maturation in the very preterm infant. In a small study of preterm infants born between 28 and 32 weeks’ gestational age (GA), structural and biochemical cerebral maturation (from 32 to 40 weeks’ GA), as determined by Magnetic Resonance Imaging (MRI), was reported to accompany neurobehaviour maturation [12]. At term equivalent age, preterm infants had less developed grey and white matter than full term infants and less mature neurobehaviour. It is well known that the period between 20–40 weeks’ GA is one characterised by rapid and vulnerable neurodevelopment [13]. Brain structural abnormalities in neonates are readily detected with MRI, and studies have reported that infants born <30 weeks’ GA have grey and white matter abnormalities [14], white matter microstructural alterations [15], deficits in brain connectivity [16], and volume reductions in other brain regions and structures [17-19]. Despite this, there has been a paucity of information to date relating the evolution of neurobehavioral alterations prior to term with cerebral alterations, especially for the most immature infants. Brain MRI during the neonatal period in preterm infants may help us to understand how early neurobehavioural development in this period relates to brain injury or structural alterations at term-equivalent age.

Furthermore, it has been suggested that modification to care practices and environmental stimulation can alter the developmental pathways of preterm infants [20]. In a small randomised controlled trial of preterm infants born between 28 and 32 weeks’ GA, Als et al. reported that modification to care practices and the environment in the NICU enhanced brain function and structure compared with standard care [20,21]. Given the potential to devise therapeutic interventions to aid development for preterm children, it is essential that we map the trajectory of neurobehavioural development prior to term age, and in particular prior to 32 weeks’ GA, in the ex-utero environment.

Alterations in neurobehaviour may manifest in a preterm infant being more irritable, taking longer to settle into a routine, and being less playful, compared with children born at term [22,23]. These child characteristics have the potential to influence the parent–child relationship, which provides the most proximal and immediate environmental context for development [24]. Characteristics of the parent also influence the parent–child relationship, with one of the most salient being parental wellbeing. Parents of infants born preterm can experience a range of responses following the birth, including symptoms of depression [25-29], anxiety [30-32], and post-traumatic stress [33]. Symptoms of depression, anxiety and stress in the post-natal period have been associated with changes in maternal behaviour such as increased maternal negativity, impairment in ability to recognise infant cues [34], reduced maternal sensitivity, increased maternal intrusiveness, and less optimal parenting behaviour [32]. Importantly, parental mental health problems have been shown to be associated with children’s later social emotional difficulties and mental health problems [35,36]. Therefore it is important that we understand more about parental wellbeing after the birth of a very preterm baby, how it changes over time, and how it relates to the parent–child relationship and child outcomes. The majority of previous studies have looked at parental wellbeing close to discharge from hospital, and usually only at one or a small number of time points, meaning that little is known about how parents adjust and cope in the first weeks after the preterm birth. In addition, the majority of studies examining parental wellbeing have focused on mothers, meaning that much less is known about paternal wellbeing following the birth of a very preterm child and how this influences children’s outcomes. Having detailed information on parental wellbeing for both parents and how it changes during the neonatal and early childhood period is important not only so appropriate supports can be implemented whilst the family is still within the hospital system and then potentially on an ongoing basis if needed, but also to know how parental psychological distress over the early years interacts with parenting beliefs and behaviours, and affects children’s outcomes. We plan to explore predictors for identifying parents who are likely to have continued psychological distress at one and two years following the birth, by examining parent characteristics such as beliefs about parenting competence, personality, previous history of mental health problems, significant life events and social risk during the neonatal period.

### Project overview

This observational study will document the evolution of neurobehavioural development in very preterm infants (defined as <30 weeks gestation) and parental psychological wellbeing during the first two years of their child’s life using serial measurements from birth. The influence of perinatal variables and parental wellbeing on the neurobehavioural pathway will be examined, along with the relationship between early neurobehaviour trajectories and MRI findings at term equivalent age and developmental outcomes at one and two years’ CA. The relationships between parental psychological wellbeing, parent–child interaction and child developmental outcomes will also be described. A control group of infants born at term will also be recruited in the neonatal period to allow comparison in outcomes and to provide a local reference group.

### Aims

The main aims of this study are to:

1. Describe the evolution of early neurobehavioural development in infants <30 weeks from birth to two years’ CA compared with children born at term, and to explore how neurobehaviour is influenced by perinatal variables.

2. Explore the relationship between early neurobehavioural development and neonatal brain abnormalities (development and injury) at term using multi-modal MRI in infants <30 weeks at birth.

3. Investigate the predictive validity of neurobehavioural assessments during the early neonatal period for developmental outcomes at one and two years’ CA in infants <30 weeks at birth.

4. Examine symptoms of depression, anxiety, and post-traumatic stress in mothers and fathers of infants <30 weeks at birth and to describe changes in parental psychological wellbeing over the first two years of the child’s life compared with children born at term.

5. Examine parental psychological wellbeing, and parent and family factors during the neonatal period as predictors of development at one and two years’ CA in children born <30 weeks, and whether these relationships are similar in children born at term.

In addition, secondary aims of this study are to:

1. Explore whether neurobehavioural examinations in the preterm period relate to concurrent physiological status (i.e. heart rate and oxygen saturations) in infants <30 weeks at birth.

2. Describe the parenting beliefs and practices of parents of infants <30 weeks at birth across the first two years of the child’s life.

3. Examine whether parental psychological wellbeing from birth to one year CA is associated with the parent–child relationship and child development when the child is one and two years’ CA in infants <30 weeks at birth.

## Methods

### Design

Prospective observational cohort study.

### Study population

Preterm infants <30 weeks’ GA at birth admitted to one of the neonatal nurseries at the Royal Women’s Hospital in Melbourne, Australia. This project aims to recruit 150 infants <30 weeks at birth over a 3-year period from January 2011. A decision was made to focus on infants <30 weeks’ GA as this is the subgroup of children considered most at-risk of developmental problems. In addition 150 term-born children will be recruited from the Royal Women’s Hospital. This study has ethics approval from the Royal Women’s Hospital Ethics Committee.

### Inclusion/exclusion criteria for preterm infants

Inclusion criteria: Infants admitted to the Royal Women’s Hospital, Melbourne, Australia, neonatal nurseries, born <30 weeks’ GA. Exclusion criteria: (i) infants with congenital abnormalities known to affect neurodevelopment and (ii) infants with non-English speaking parents.

### Inclusion/exclusion criteria for term-born infants

Inclusion criteria: Infants admitted to the Royal Women’s Hospital Melbourne, Australia, born >36 completed weeks’ GA and weighing >2500 g. Exclusion criteria: (i) infants with congenital abnormalities known to affect neurodevelopment (ii) infants requiring admission to neonatal intensive or special care nursery and (iii) infants with non-English speaking parents.

### Recruitment

A research nurse will approach eligible families of very preterm children within the first or second week of life, following approval from the medical team. The research nurse will verbally explain the study, including the time commitment and give written information on the study. Both parents will be invited to be in the study. If the parent/s agree to be in the study they will be asked to sign a consent form. Families of term-born infants will be approached by a research nurse prior to discharge following the same methodology as above.

### Perinatal data collection

Following consent, the research nurses will collect maternal and perinatal data that are known to be related to neonatal and long-term outcome from medical histories. These data include pregnancy complications (e.g. pre-eclampsia) and treatments (e.g. magnesium sulphate), birth weight, sex, GA at birth, significant neonatal complications including grade of intraventricular haemorrhage, cystic periventricular leukomalacia, necrotising enterocolitis, culture positive infections and chronic lung disease, and the need for postnatal corticosteroids. A family questionnaire will be used to assess various sociodemographic factors including *The Social Risk Index*[37], which assesses 6 aspects of social status including family structure, education of primary caregiver, occupation of primary income earner, employment status of primary income earner, language spoken at home, and maternal age at birth. Also incorporated in this questionnaire will be items assessing parental alcohol and drug use, and mental health service access history which will be completed individually by mothers and fathers where possible.

## Child assessments

### Serial neurobehavioural assessments up to term (preterm infants only)

There is no single assessment tool, with good reliability, that has been validated for use prior to term equivalent age to assess neurobehaviour from birth [2]. Therefore, we will use four assessment tools in this study, the NICU Network Neurobehavioral Scale (NNNS) [38], the Hammersmith Neonatal Neurological Examination (HNNE) [39], Prechtl’s assessment of General Movements (GMs) [40] and the Premie-Neuro (Table 1) [41]. The NNNS and HNNE have been validated for use at term equivalent age, however, there are many items that are not appropriate for more preterm and medically unstable infants, for example “pull to sit” or “following an object”. We will therefore need to exclude or modify these items for the assessment of infants at earlier GAs. GMs and the Premie-Neuro are appropriate for use from preterm birth and will not be modified. In addition to these standardised assessments, we will develop a new assessment tool to measure neurobehaviour, which will involve observation of the infant’s behavioural cues during regular care procedures and the neurobehavioural assessments.

**Table 1:** Description and purpose of neurobehavioural assessments

**Assessment**	**Purpose**
NNNS [38]	The *Neonatal Intensive Care Unit Network Neurobehavioral Scale* (NNNS) assesses the neurological integrity, behavioural functioning, and responses to stress in high-risk infants using 45 items compared with norms for healthy term infants (n = 125). The NNNS provides an in-depth assessment of neurobehaviour and gives summary scores/subscales for attention, handling, quality of movement, regulation, nonoptimal reflexes, arousal, hypertonicity, hypotonicity, asymmetrical reflexes, excitability and lethargy.
HNNE [39]	The *Hammersmith Neonatal Neurologic Examination* (HNNE) consists of 34 individual items with 6 subtotals including tone, tone patterns, reflexes, spontaneous movements, abnormal neurological signs and behaviour in newborns. It provides an overall “optimality score” which has been validated in healthy term (n = 224) and preterm (n = 380) infants. This assessment tool is used frequently in clinical practice and requires no formal training.
GMs [40]	Prechtl’s *General Movements* (GMs) assessment is a non-invasive method for assessing global neurological development, particularly motor development. Video recordings are made of spontaneous whole body movements and assessed at a later time by independent assessors. GMs during the neonatal period have been shown to be predictive of cerebral palsy from birth in the preterm infant. This assessment has the advantage of obtaining an overall picture of neurological integrity without needing to handle the infant.
Premie-Neuro [41]	The Premie-neuro is a brief neurological examination for preterm infants aged 23–37 weeks’ gestation. Consists of 24 items divided into neurological, movement and responsiveness subgroups. Validity has been shown in a small study (n = 34), however the inter-rater reliability was low.

Each examination will be video-recorded so that assessments can be scored later as appropriate. As infants prior to term age in the NICU can be sensitive to handling we will not administer any item considered to cause the infant unnecessary stress. During the assessment, we will simultaneously observe pulse oximetry data, which will give information on oxygen saturations and heart rate. If these data and other behaviour, such as apnoea, suggest increased stress levels in the infant as a result of the neurobehavioural assessment, administration of that item will cease. The assessment will be continued where possible. Due to the poor self-regulation abilities and sensitivity to handling of infants younger than 30 weeks’ GA, it will be necessary for early evaluations (<30 weeks’ GA) to principally involve observation, focusing on: GMs [40], state, motor and autonomic regulation and the infant’s response to external stimuli. Behavioural (e.g. colour changes, facial expressions) and motor observations (e.g. postural tone and quality of spontaneous movements) will be video-recorded during a standard care procedure (e.g. a nappy change) to evaluate the infant’s response to handling during an everyday activity. The video recordings will allow the assessor to make more detailed assessments than they are able to do in real-time and allow for inter-observer reliability of scoring (two scorers will be used to assess reliability). Evaluations will be completed weekly from enrolment up to 32 weeks’ GA, then fortnightly until term, or discharge from the Royal Women’s Hospital. Assessments will be timed to coincide with care procedures to ensure the infants’ sleeping patterns are not disrupted. Initially assessments will take approximately 15 minutes and increase to 30 minutes as the infants become older and more stable, enabling us to elicit more specific responses.

To standardise the assessment procedure in the neonatal nursery, we will assess the infant in an environment with minimal lighting and low noise levels. The assessors will be health professionals (e.g. physiotherapists, occupational therapists, nurses and physicians) who have received training in the neurobehavioural assessment and who are not involved with the clinical care of the child. All assessors will have accreditation for the relevant assessment tools where required (i.e. NNNS and GMs). The assessors will liaise closely with the clinical team, particularly the bedside nurse, to time the assessment with the baby’s care to minimise handling.

### Assessment at term equivalent age (both preterm and term infants)

#### Serial neurobehavioural assessments

At term equivalent age (38–44 weeks’ GA) infants will have neurobehavioural assessments carried out by an independent assessor blinded to previous examination results and clinical history (including prematurity). The term neurobehavioural assessment will consist of the NNNS, followed by the additional items needed to complete the HNNE (i.e. reflexes, arm and leg recoil) and five minutes of video footage for GMs of the infant in an active or quiet alert state.

#### Magnetic resonance

The MRI scan is not a compulsory component of the study and parents consenting to the study can choose for their baby not to have the MRI scan. For those who consent, MRI scans will be performed without anaesthesia or sedation, between 38–44 weeks’ GA on the same day as the term equivalent age neurobehavioural assessment. All scans will be performed at the Royal Children’s Hospital using the 3 T Siemens Magnetom Trio MRI scanner. The scanning session will take approximately 60 minutes. A full anatomic, functional, developmental and metabolic infant brain MRI study will be performed using the following sequences:

•**T2-weighted images:** Transverse Restore turbo spin echo imaging: Flip angle = 120, Repetition Time = 8910 ms, Echo Time = 152 ms, Field Of View = 192 × 192 mm, Matrix = 192 × 192, 1 mm [3] isotropic voxels.

•**T1-weighted images:** Transverse multi-planar reconstruction imaging with noise suppression: Flip angle = 9°, Repetition Time = 2100 ms, Echo Time = 3.39 ms, Field Of View = 192 × 192 mm, Matrix = 192 × 192, 1.0 mm [3] isotropic voxels.

•**Diffusion weighted imaging:** Transverse echo planar imaging: Repetition Time = 20400 ms, Echo Time =120 ms, Field Of View =173 × 173 mm, Matrix = 144 × 144, 1.2 mm [3] isotropic voxels, 45 gradient directions (range b = 100 to b = 1200s/mm [2]), 3 b = 0 s/mm [2].

•**Resting state functional connectivity MRI:** Transverse 2D echo planar imaging with prospective acquisition correction: Repetition Time = 2910 ms, Echo Time = 28 ms, flip angle = 90°, Field Of View = 151 × 151 mm, Matrix = 64 × 64, 2.4 mm [3] isotropic voxels.

•**Proton magnetic resonance spectroscopy:** Transverse spin echo chemical shift imaging: Repetition Time = 2000, Echo Time = 135, flip angle = 90°, scan resolution = 12 × 12, interpolated 16 × 16, field of view = 103 × 125 mm, voxel size = 10.4 × 8.6 × 15.0 mm.

The MRI scans will be qualitatively evaluated by two independent investigators who are blinded to the clinical neurobehavioural assessment, utilising an established scoring method for newborn infants [42]. This scoring system provides an overall measure of the presence and severity of white matter, cortical grey matter, deep grey matter and cerebellar abnormalities (recorded as normal, mild, moderate, or severe). Quantitative image analysis will involve the following image analyses:

•**Structural T1-weighted and T2-weighted MR image analyses:** Grey and white matter and cerebrospinal fluid will be initially segmented using SPM8 software (http://www.fil.ion.ucl.ac.uk/spm/software/spm8) with tissue priors from a 40-week neonatal template [43], and then fed into a modified version of a previously published morphology-driven automatic segmentation pipeline [44] to obtain volumes of white matter, cortical grey matter, cerebrospinal fluid, deep nuclear gray matter, brainstem, hippocampus, amygdala and cerebellum [19]. These assessments will enable an assessment of size and morphological alterations to global and regional areas of the brain.

•**Diffusion imaging and tractography:** Vulnerable white matter fibre bundles of the visual, motor, language and attention pathways will be isolated by tractography. Diffusion tractography enables the characterisation of particular fibre tract populations which can then be related to early neurobehavioural functions. Tract-specific diffusion measures of fractional anisotropy, mean diffusivity, axial and radial diffusivity will be calculated. Diffusion measures provide insight into white matter tissue integrity reflecting microstructural organisation, water content, number and density of axons, and myelination, and are useful for gauging white matter maturity [45,46]. Structural connectivity will also be performed, where white matter fibre networks will be analyzed using graph theory metrics [47].

•**Resting state functional MRI:** Resting state functional connectivity (fcMRI) will be assessed by detecting temporal correlations in spontaneous blood oxygen level dependent (BOLD) signal oscillations while subjects rest quietly in the scanner. Distinct resting-state networks related to vision, language, executive processing and other sensory and cognitive domains will be identified. This will allow relationships between specific neurodevelopmental impairments and functional brain networks to be determined [48].

•**Proton magnetic resonance spectroscopy:** Brain metabolites in specific brain regions will be measured using proton MR spectroscopy. Measurements will be made for (a) N-acetyl aspartate, which is reduced as a result of destruction of neurons or decreased neuronal integrity (b) Lactate, which is elevated in regions where cell and tissue necrosis have occurred (c) Choline which reflects cellularity and is elevated in response to demyelination and gliosis (d) Glutamine and glutamate, markers for neuronal damage, and (e) myo-inositol, a marker for myelin breakdown. Levels of these markers, in regions of interest from the frontal and occipital white matter will be important to elucidate the association between neurobiological abnormalities as a result of hypoxic ischaemic events or infection/inflammation and adverse early neurobehavioural characteristics.

### Outcomes at one and two years’ corrected age (both preterm and term infants)

At one and two years’ CA families will be contacted to participate in follow-up. The child will have a developmental assessment, both parents will be asked to participate (separately) in a parent–child interaction task, and both parents will be asked to complete a set of questionnaires.

#### Developmental assessment

A range of developmental outcomes will be assessed at one and two years’ CA by a blinded assessor (Table 2). At one year, motor development will be assessed by the Alberta Infant Motor Scale (AIMS) [49] and the Neuro-Sensory Motor Developmental Assessment (NSMDA) [50], neurological outcomes with the Touwen Infant Neurological Examination (TINE) [51] and oral motor development with the Schedule for Oral Motor Assessment (SOMA) [52] (Table 2). The AIMS, NSMDA and TINE will be administered by a physiotherapist or occupational therapist and will take approximately 30 minutes. For the SOMA, infants will be seated in a high chair and food and fluid trials will be offered by a research nurse trained in the procedure or by a speech-language pathologist using the standard administration approach. Infants will only be offered food categories that they are currently managing in the home environment. Parents will be instructed to use the standard administration approach in instances where infants refuse to eat for the research nurse or speech pathologist. The SOMA takes approximately 20 minutes to administer and will be videotaped for later rating of the infants’ oral-motor skills by a speech-language pathologist who is blinded to the child’s clinical history.

**Table 2:** Description and purpose of neurodevelopmental assessments

**Assessment**	**Purpose**
AIMS [49]	The *Alberta Infant Motor Scale* (AIMS) is an observational norm-referenced assessment that measures infant motor development between 0 to 18 months of age. There are 58 items across the four positional subscales of prone, supine, sit and stand. The infants least and most mature item in each subscale is identified and marked as observed, then a window is created to assess the items in between as either observed or not observed. Subscale scores are added to obtain a total score. This assessment has been used extensively in follow-up of preterm infants and has excellent psychometric properties [53].
NSMDA [50]	The *Neuro-Sensory Motor Developmental Assessment* (NSMDA) is a criterion-referenced assessment tool constructed to measure neurodevelopment between 1 month and 6 years of age. The five domains of neurological, postural, sensory, fine, and gross motor are summed to create a total NSMDA score. A functional grade is also given for each domain and totalled to provide a total functional grade of normal, minimal deviation, mild deviation, moderate deviation, severe deviation or profound deviation. The NSDMA has good predictive validity for long term motor development [54].
TINE [51]	The *Touwen Infant Neurological Examination* (TINE) is a neurological examination designed for use with infants post term age. There are five clusters of dysfunction assessed – reaching and grasping, gross motor development, brainstem, visuomotor and sensorimotor. The number of criteria fulfilled is recorded for each cluster with an overall dysfunctional cluster rating of yes or no determined. The number of dysfunctional clusters are then added together to determine a neurological classification of neurologically normal, normal sub-optimal, MND (minor neurological dysfunction) or abnormal. The TINE has been shown to predict both minor and major neurological dysfunction in at risk populations including preterm infants [55].
SOMA [52]	The *Schedule for Oral Motor Assessment (SOMA)* is a standardised and psychometrically robust measure of oral-motor skills for eating and drinking for infants aged 8 months to 2 years. It was designed primarily to assess a wide range of oral-motor skills in infants with a grossly intact neurological system.
Bayley-III [56]	The *Bayley Scales of Infant and Toddler Development 3*^*rd*^*edition* (*Bayley-III)* is a norm referenced developmental scale of cognitive, language and motor development that has good psychometric properties when used with a local control group, and has been used extensively in the follow-up of preterm infants [53,57].

At two years’ CA, development will be assessed using the Bayley Scales of Infant and Toddler Development – 3^rd^ edition (Bayley-III) [56]. A neurological paediatric assessment will also be performed by a trained paediatrician to assess for cerebral palsy, as well as other sensory problems, such as blindness or deafness. The assessment at two years will take approximately 2 hours.

#### Parent–child relationship assessment

The parent–child relationship for both preterm and term born infants will be assessed using the Emotional Availability Scales (EAS) 4^th^ edition [58]. The EAS is an observational measure examining the contribution of the parent and the child to the parent–child relationship in terms of their emotional responsiveness and attunement to the other member of the dyad. The measure consists of six global emotional availability dimensions – adult sensitivity, adult structuring, adult non-intrusiveness, adult non-hostility, child responsiveness and child initiation. Parents and children will be videotaped in semi-structured play interactions for approximately 15 minutes. The interactions will be coded at a later time by fully trained and accredited coders. The EAS has been used widely with different populations and has evidence of good reliability and validity. For example, the EAS has been associated with other measures of attachment in the parent–child relationship [59] and child development [60], and is reliable across contexts [61].

Table 3 provides an overview of the child assessments and parent-child interaction assessments to be collected over the first two years.

**Table 3:** Administration timetable for infant assessments

	**Birth**	Up to 32wk*	32wk to term**	**Term**	**12mth (CA)**	**24mth (CA)**
Neurobehavioural assessment	√	√	√	√√		
Perinatal data	√√					
MRI				√√		
Motor assessment – AIMS and NSMDA					√√	
Clinical feeding assessment - SOMA					√√	
Developmental assessment – Bayley III						√√
Parent–child relationship assessment- EAS					√√	√√
Neurological and Paediatric assessment						√√

*weekly assessments; **fortnightly assessments; √√ = both preterm and term-controls; √ = preterm only; MRI = Magnetic resonance imaging; AIMS = Alberta Infant Motor Scale; NSMDA = Neuro-Sensory Motor Developmental Assessment; SOMA = Schedule for Oral Motor Assessment ; Emotional Availability Scales.

#### Parental assessment

Parent questionnaires will be collected at multiple time points. The first will be when their infant has their first neurobehavioural assessment. Both parents will then be asked to complete questionnaires fortnightly until term equivalent age, at term equivalent age, at three and six months, and at six-monthly intervals until the child’s second birthday (CA). The questionnaires at 3, 6 and 18 months will be posted to families, and asked to be returned to the investigators in a prepaid envelope. Parents will be sent text messages as a reminder to complete questionnaires. The term, one and two year questionnaires will be completed at the time of the infant’s follow-up assessments. Not all measures will be collected at all time points. Each questionnaire to be used in the study is described in Table 4. The time taken to complete these questionnaires will vary from 5 minutes at earlier ages to approximately 2 hours (total) at 24 months’ CA.

**Table 4:** Description and purpose of parental questionnaires

**Assessment**	**Purpose**
CES-D	The *Centre for Epidemiological Studies Depression Scale (CES-D)*[62] will be used to measure depressive symptoms. The questionnaire consists of 20 questions (total score range 0 to 60) with higher scores representing greater depressive symptoms. A score ≥16 on the CES-D represents significant depressive symptoms. This threshold has been shown to correlate well with clinician ratings of depression [63]. The CES-D has been used extensively in general populations and has been used with parents of preterm infants [64,65].
HADS	The *Hospital Anxiety and Depression Scale (HADS)*[66] will be used to assess anxiety. The HADS assesses symptoms of anxiety and depression using 7 items for each scale that are scored with a 4-point rating scale (total score range 0 to 21). Scores above 11 are considered to indicate significant symptoms of depression or anxiety. The HADS has been validated in a variety of settings and has been found to perform well in assessing the severity of anxiety disorders and depression, not only in primary care patients and the general population [67] but also in parents of preterm infants [68].
PCL-S	The *Posttraumatic Stress Disorder Checklist Specific Version*[69] (PCL-S) will be used to assess symptoms of posttraumatic stress disorder (PTSD). The PCL-S consists of 17 items (total score range 17 to 85). The questions are asked in relation to a nominated specific traumatic event, in this case, the birth of their very preterm infant. There is evidence for good test-retest reliability, internal consistency and convergent validity [70]. Only parents of preterm infants will complete the PCL-S.
IPIP-NEO	Neuroticism, or negative affectivity, will be measured with the 10-item Neuroticism scale of the *International Personality Item Pool Five Factor Personality Inventory* (IPIP-NEO) [71]. The Neuroticism scale selected for the present study is from a 50-item self-report version of the NEO PI-R, named the IPIP-NEO [72]. Responses on the Neuroticism scale are scored on a 5-point scale.
PSOC	The *Parenting Sense of Competence Scale* (PSOC) assesses parental satisfaction and efficacy in the parenting role, with higher scores representing higher satisfaction and efficacy in parenting. It is a 16 self-report measure with each item rated by parents on a 6-point rating scale. The PSOC has been widely used and there is good evidence for the validity of the measure [73].
CISS	The *Coping Inventory of Stressful Situations* (CISS) is a 48-item inventory which will be used to measure three major types of coping styles in an individual, including Task-Oriented (problem-solving), Emotion-Oriented (focuses on consequent emotions, becoming angry/upset), and Avoidance Coping (distraction and social diversion) [74]. Parents will be asked to rate each item on a 5-point scale ranging from (1) “not at all” to (5) “very much”.
PSI-LSS	The *Life Stress Scale* from the *Parenting Stress Index (PSI-LSS)*[75] assesses how many of 19 significant life events have occurred for parents within the last 12 months such as divorce, went deeply into debt, entered new school.
Demographic & family questionnaire	Items include relationship to child, whether the parent is the primary caregiver, number of other children in the home, cultural background.
Parent mental health history questionnaire	Five items will assess parental cigarette, alcohol and recreational drug use, and mental health service access history.
Parenting practices questionnaire	The *Parenting Practices Questionnaire* is a 16-item measure that assesses parental warmth, hostility and involvement with their child (Longitudinal Study of Australian Children, LSAC).
ITSEA	The *Infant Toddler Social and Emotional Assessment*[76] (ITSEA) is a 135 item parental report measure of social–emotional problems and competencies in 1 to 3 year olds. It assesses 4 broad domains of behaviour: dysregulation, externalizing, internalizing and competencies. Mean scores below the 10^th^ percentile for competence, or above the 90^th^ percentile for externalising, internalising and dysregulation suggest the infant may be at risk for psychopathology. The ITSEA has good internal consistency, validity, and test-retest reliability, and has been used extensively, including with very preterm populations.
MacArthur CDI	The *MacArthur-Bates Communicative Development Inventories (CDI)*[77] are standardised parent report forms for assessing early language (semantic and grammatical) development in 16 to 30 month old children. The CDI: Words and Sentences (Toddler form) will be completed by the primary care-giver.
FAD	The *Family Assessment Device (FAD)*[78] requires the primary caregiver to indicate whether they “strongly agree”, “agree”, “disagree”, or “strongly disagree” with 60 statements about family functioning. The inventory yields 7 scales: problem solving, communication, roles, affective responsiveness, affective involvement, behaviour control and general functioning. Higher scores indicate poorer family functioning.
ITSP	The *Infant/Toddler Sensory Profile Questionnaire* (ITSP) [79] consists of 48 questions, addressing 6 sensory processing sections, including: Auditory, Visual, Tactile, Vestibular, and Oral Sensory Processing, as well as a General measure. Questions within each sensory processing section yield information about how the child responds to stimuli in each sensory system. Its purpose is to evaluate the possible contributions of sensory processing to the child’s daily performance patterns, to provide information about his or her tendencies to respond to stimuli and to identify which sensory systems are likely to be contributing to or creating barriers to functional performance. The ITSP has excellent content validity and adequate to excellent reliability [80].
CSBS-DP	The *Infant-Toddler checklist* from the *Communication and Symbolic Behavior Scales Developmental Profile* (CSBS-DP) [81] is a 24 item screening tool designed to measure 7 predictors of language including emotion and eye gaze, communication, gestures, sounds, words, understanding and object use in children aged between 6 and 24 months of age.
Social Risk Index	A *Social Risk Index* (family demographic questionnaire) [37] score will be calculated based on a combination of family structure, education of primary caregiver, employment of primary income earner, language spoken at home and maternal age at the birth of the child. Higher scores indicate higher social risk.

Table 5 provides an overview of the questionnaire measures to be collected at each time point. For term-born infants, birth and term are the same time point in Table 5.

**Table 5:** Administration timetable for parent completed questionnaires

	**Birth**	F/night*	**Term**	**3mth (CA)**	**6mth (CA)**	**12mth (CA)**	**18mth (CA)**	**24mth (CA)**
CES-D	√	√	√√	√√	√√	√√	√√	√√
HADS	√	√	√√	√√	√√	√√	√√	√√
PCL-S			√			√		√
IPIP-NEO	√							
PSOC					√√	√√		√√
PSI-LSS	√√					√√		√√
CISS	√√				√√			
Demographic & family questionnaire	√√							
Parent mental health history questionnaire			√√			√√		√√
Parenting practices					√√	√√		√√
ITSEA						√√		√√
MacArthur CDI								√√
FAD								√√
ITSP								√√
CSBS-DP								√√
Social Risk Index	√√					√√		√√

Note. *F/night = administered fortnightly from first neurobehavioural assessment until term equivalent age; √√ = both preterm and term-controls; √ = preterm only; CES-D = Centre for Epidemiological Studies Depression Scale; HADS = Hospital Anxiety and Depression Scale; IPIP-NEO = International Personality Item Pool Five Factor Personality Inventory-NEO, PCL-S = Posttraumatic Stress Disorder Checklist Specific Version; CISS = Coping Inventory for Stressful Situations; PSOC = Parenting Sense of Competence Scale; PSI-LSS = Parenting Stress Index – Life Stress Scale; ITSEA = Infant-Toddler Social and Emotional Assessment, MacArthur CDI = MacArthur Communicative Development Inventories, FAD = Family Assessment Device, ITSP = Infant Toddler Sensory Profile, CSBS – DP = Communication and Symbolic Behavior Scales Developmental Profiles.

#### Data collection & analysis

The data will be analysed according to the following main aims:

1. To describe the longitudinal evolution of early neurobehavioural development in very preterm infants (<30 weeks’ GA), from birth to two years CA the mean and 95% confidence intervals (CI) for the neurobehavioural assessment will be presented by week up to 32 weeks’ gestation, then fortnightly from 32 weeks’ gestation to term equivalent age, as well as at 1 and 2 years corrected age. Data will be presented according to both gestational and chronological age. Trends over time (according to both chronological and gestational age) will be explored using a mixed effects regression model for each outcome fitted to the continuous age measurement with a fixed effect of time (age) and a random effect for individuals to allow for the repeated observations on each infant.

2. To explore whether the neurobehavioural trajectory varies according to MR findings at term, qualitative MRI variables of interest will be dichotomised into two distinct categories (moderate to severe vs. none or mild white matter injury). The mean (and 95% CI) scores for the neurobehavioural development outcomes at each time point (as described for aim 1) will be plotted separately for children within each category. The change in neurobehavioural development over time (according to both chronological and gestational age) will be modelled using separate mixed effect regression models for each of the neurodevelopmental scores with a fixed effect of time (age) an indicator for moderate-severe injury and an interaction between time and the injury indicator, to assess whether the effect of time is different in those with a moderate-severe injury compared with those with no or a mild injury. The association between early neurobehavioural development and quantitative MRI (e.g. volumes, diffusion, metabolites such as NAA and cho) will be analysed using linear (continuous outcomes) and logistic (binary impairment outcomes) fitting a separate regression model for each outcome and neurobehaviour time point (according to both chronological and gestational age).

3. The validity of early neurobehavioural assessments from birth to term-equivalent age for predicting development at one and two years’ CA in very preterm children will be investigated using separate linear (continuous outcomes) and logistic (binary impairment outcomes) regression models for each neurobehavioural assessment time point according to chronological and gestational age and each outcome. Estimates of the regression coefficients and the R^2^ values (representing the proportion of variability in the outcome explained by the model) from these models will be compared with estimates from the same models using neurobehaviour at term equivalent age as the predictor to obtain an idea of the predictive ability of these early measurements compared with the measurements at term as used currently. Data from term-born controls will be used to provide a local-reference population for calculation of mild, moderate and severe developmental impairment (mild = more than 1 SD below the mean, moderate = more than 2 SD below the mean, severe = more than 3 SD below the mean on the Cognitive Composite Scale on the Bayley-III).

4. To examine symptoms of depression, anxiety, and post-traumatic stress in mothers and fathers of very preterm children at birth during the first two years of the child’s life compared with term-born controls, we will present means and 95% CI for each parental assessment at each time point. For each time point, the observation closest to the specified time for the completed week within +/− 3.5 days for weekly assessment and +/− 7 days for fortnightly assessments will be selected for each individual.

5. To examine the relationships between parental psychological wellbeing, parent and family factors, and very preterm children’s neurobehavioural development at birth, and later two year-old cognitive, motor and social-emotional developmental outcomes, we will fit separate linear regression models for each of the two year outcomes. Initially each parent and child factor will be explored using separate univariable models for each outcome before combining important factors into a single model for each outcome to assess independent predictors.

### Secondary aims

1. To explore whether the neurobehavioural trajectory varies according to concurrent physiological status, physiological status variables of interest will be dichotomised or separated into distinct categories (e.g. stable versus unstable). The mean (and 95% CI) scores at each time point (as described above) will be plotted separately for children within each category. Trends over time will be modelled using separate mixed effects regression models for each of the neurobehavioural scores including a fixed effect for time (gestational and chronological age) and physiological status and an interaction between time and physiological status to assess whether the effect of time is different in the different categories of this variable.

2. To describe the parenting beliefs and behaviour of parents of very preterm children across the first two years of the child’s life compared with term-born controls, we will present means and 95% CI for each parental assessment at each time point from term equivalent age to two years. Trends over time will be compared between the two groups using separate mixed effects regression models for each parent, with a fixed effect of time (gestational and chronological age) and group as well as an interaction between time and group. Models will also include a random effect to allow for repeated measures within a parent.

3. To examine whether parental psychological wellbeing is associated with the parent–child relationship when the child is one and two years’ CA, average scores for parental mental health from birth to 6 months, and from one to two years will be used as predictors of the 6 outcomes scales of the parent–child relationship measure at one and two years CA respectively. Separate linear regression models will be used for mothers and fathers to examine these relationships.

### Sample size

A sample of 150 very preterm infants and 150 term-born controls was chosen based upon projected workload, the number of infants <30 weeks cared for each year at the Royal Women’s Hospital, and the expected consent rate for the study from families. A sample of 150 infants will enable us to estimate the mean of any neurobehavioural measure employed in this research at any time point to within +/−0.16 standard deviations (based on a 2-sided 95% confidence interval, Aim 1), and to identify correlations between observations as small as 0.2 (Aims 2 and 3), with 80% power (based on 5% significance).

## Discussion

This protocol outlines a comprehensive longitudinal study of early neurobehavioural and parental psychological wellbeing of infants born <30 weeks’ GA and their families. A trajectory of development for these individuals and their families would enable us to determine (i) when and how alterations in neurobehaviour occur, (ii) whether there are specific patterns of neurobehaviour (iii) how the development of neurobehaviour development relates to perinatal factors, neonatal interventions and the intensive care environment, (iv) how the development of neurobehaviour relates to brain abnormalities detected by MRI at term, and (v) how early patterns of neurobehaviour relate to development later in childhood. This information will be invaluable for the planning and implementation of future rehabilitation projects aimed at minimising neurobehavioural deficits prior to term equivalent age. Currently there are no such published norms on the early neonatal neurobehaviour of very preterm infants. Furthermore, this will be the first study to screen for parental symptoms of depression, anxiety and post-traumatic stress at multiple time points in both mothers and fathers to create a detailed trajectory of parental psychological wellbeing following very preterm birth. This will allow us to (i) explore the psychological wellbeing of both mothers and fathers, (ii) incorporate a longitudinal design with multiple assessment time points during the hospitalisation and post-discharge periods, and (iii) investigate the implications of parental psychological distress on parent–child interaction and child development. A key feature of this study is the focus on fathers, as symptoms of distress in fathers following preterm birth have been largely neglected, especially with high quality longitudinal research of this nature.

## Competing interests

The authors declare that they have no competing interests.

## Author’s contributions

AJS, DKT, NBC, KT, JLYC, KJL, CCP, JO, LA, ATM, AE, MS, FJ, LWD and PJA have made substantial contributions to conception and design of this study. AJS, DKT, NCB, CCP, JO, LA and AE have had a role in the acquisition of data. All authors have been involved in drafting the manuscript or revising it critically for important intellectual content and have given final approval of the version to be published.

## Pre-publication history

The pre-publication history for this paper can be accessed here:

http://www.biomedcentral.com/1471-2431/14/111/prepub
